# Clinical Immunogenicity of DaxibotulinumtoxinA for Injection in Glabellar Lines: Pooled Data from the SAKURA Phase 3 Trials

**DOI:** 10.3390/toxins15010060

**Published:** 2023-01-10

**Authors:** Conor J. Gallagher, Ronald R. Bowsher, Amanda Clancy, Jeffrey S. Dover, Shannon Humphrey, Yan Liu, Gregg Prawdzik

**Affiliations:** 1 Revance Therapeutics, Inc., Nashville, TN 37203, USA; 2 B2S Life Sciences, Franklin, IN 46131, USA; 3 SkinCare Physicians, Chestnut Hill, MA 02467, USA; 4 Department of Dermatology and Skin Science, University of British Columbia and Humphrey Cosmetic Dermatology, Vancouver, BC V5Z 4E1, Canada

**Keywords:** immunogenicity, neutralizing antibodies, botulinum toxins, type A, DAXI, daxibotulinumtoxinA, excipients, neuromodulator, peptides

## Abstract

DaxibotulinumtoxinA for Injection (DAXI) is a novel botulinum toxin type A product containing daxibotulinumtoxinA with a stabilizing excipient peptide (RTP004). DAXI immunogenicity was assessed in three phase 3 glabellar line studies (two placebo-controlled, single-dose studies and an open-label repeat-dose safety study). Binding antibodies to daxibotulinumtoxinA and RTP004 were detected by validated ELISAs. Samples positive for daxibotulinumtoxinA-binding antibodies were evaluated further for titer and neutralizing antibodies by mouse protection assay. Overall, 2786 subjects received DAXI and 2823 subjects were exposed to RTP004 as DAXI (*n* = 2786) or placebo (*n* = 37). Treatment-related anti-daxibotulinumtoxinA binding antibodies were detected in 21 of 2737 evaluable subjects (0.8%). No subject developed neutralizing antibodies. Treatment-related anti-RTP004 binding antibodies were detected in 35 (1.3%) of 2772 evaluable subjects. Binding antibodies were generally transient, of low titer (<1:200), and no subject had binding antibodies to both daxibotulinumtoxinA and RTP004. All subjects with treatment-induced binding antibodies to daxibotulinumtoxinA or RTP004 achieved none or mild glabellar line severity at Week 4 following each DAXI cycle, indicating no impact on DAXI efficacy. No subjects with binding antibodies to daxibotulinumtoxinA or RTP004 reported immune-related adverse events. This evaluation of anti-drug antibody formation with DAXI shows low rates of antibody formation to both daxibotulinumtoxinA and RTP004.

## 1. Introduction

Commercial preparations of botulinum toxin type A (BoNTA), generally derived from the Hall strain of Clostridium botulinum, have a variety of therapeutic applications, such as the treatment of cervical dystonia, upper limb spasticity, chronic migraine headaches, overactive bladder, and blepharospasm, and are widely used for aesthetic treatment of facial lines. In these indications, the product is administered via injection directly into striated or smooth muscle or dermis guided by electromyography (EMG) or ultrasound or by reference to surface anatomical landmarks. BoNTA effects are based on their highly specific and well-characterized ability to block cholinergic innervation of striated and smooth muscle, and cholinergic autonomic innervation of exocrine glands. However, as with all macromolecular biotherapeutics, BoNTAs have the potential to be immunogenic and provoke the formation of unwanted anti-drug antibodies [1]. Therefore, the evaluation of unwanted immunogenicity is an integral component in the overall clinical safety assessment of new candidate BoNTAs. In addition to safety, this regulatory requirement typically includes an investigation of the potential consequences of anti-drug antibody formation on drug efficacy and pharmacokinetics.

With respect to BoNTA therapeutics, anti-drug antibodies can potentially form to any portion of the protein complex, including the active 150-kDa core neurotoxin [2,3] and the hemagglutinin and non-hemagglutinin neurotoxin-associated proteins (NAPs), which form stabilized complexes with the core neurotoxin in vivo [4]. Importantly, neutralizing antibodies can block the biological activity of the BoNTA, typically by recognizing specific epitopes at the C-terminus of the 100-kDa heavy chain, the area known to bind the cognate receptors for BoNTA [2]. Consequently, this type of anti-drug antibody has the capability to interfere with the therapeutic’s desired pharmacology, whereas non-neutralizing binding antibodies do not interfere with the activity of the neurotoxin, and efficacy is unaltered [5]. 

DaxibotulinumtoxinA for Injection (DAXI) is a novel, highly purified BoNTA product formulated with a unique proprietary protein transduction domain (PTD)–containing excipient. Produced following the fermentation of Clostridium botulinum, the neurotoxin undergoes multiple purification steps, including three-column chromatography steps, to minimize the presence of residual bacterial proteins or genetic material and to remove the NAPs, leaving a purified 150-kDa core neurotoxin (daxibotulinumtoxinA). In place of the human serum albumin typically found in commercial BoNTAs, DAXI is stabilized with a novel proprietary peptide excipient (RTP004). RTP004 is a synthetic 5-kDa, 35 amino acid (AA) polypeptide (RKKRRQRRRGKKKKKKKKKKKKKKKGRKKRRQRRR) composed of a 15 AA poly-lysine core with a 9 AA PTD at either end, spaced by a linker amino acid. The PTD is modeled on the sequence first identified in the 100 AA transactivator of transcription (TAT) protein sequence. To our knowledge, DAXI is likely to be the first therapeutic product containing a PTD to be approved by any regulatory agency. 

RTP004 is highly positively charged and binds non-covalently, but tightly, to the negatively charged core neurotoxin [6], stabilizing the neurotoxin molecule to prevent protein aggregation and adsorption of the neurotoxin to charged surfaces [7]. The strong net positive charge of RTP004 also drives electrostatic binding to negatively charged neuronal surfaces and extracellular matrix proteins [8,9] and may facilitate increased internalization of the neurotoxin. This, in turn, may explain why the median duration of effect observed clinically with DAXI is longer than has been reported for other BoNTAs in therapeutic [10,11,12,13] and aesthetic indications [14,15,16,17,18] despite DAXI containing a similar or lower amount of core neurotoxin per dose [13]. This clinical benefit could also potentially reduce immunogenic risk by allowing less frequent retreatment. DAXI is manufactured without the use of animal products, is highly purified, and does not contain accessory proteins other than the synthetic excipient RTP004; however, the clinical immunogenicity of RTP004, or any PTD-containing drug product, has not been previously reported.

This article presents a comprehensive evaluation of DAXI’s immunogenicity profile following repeated administration in adults enrolled in the extensive DAXI clinical program for the treatment of glabellar lines. Based on the high degree of purity of DAXI and the low rates of immunogenicity identified with other BoNTA products, we hypothesized the overall degree of antibody formation to DAXI to be very low. Detection and characterization of serum anti-drug antibody responses against both the daxibotulinumtoxinA neurotoxin and the novel excipient peptide RTP004 were performed using validated enzyme-linked immunosorbent assays (ELISAs). Combining data from three phase 3 studies—SAKURA 1, 2, and 3—provided a unique opportunity to evaluate DAXI immunogenicity after multiple treatments in a large population, to evaluate the temporal relationship between treatment and antibody development, and to evaluate whether RTP004 exerted an effect on daxibotulinumtoxinA immunogenicity.

## 2. Results

### 2.1. Subject Disposition and Baseline Characteristics

**Figure 1:**
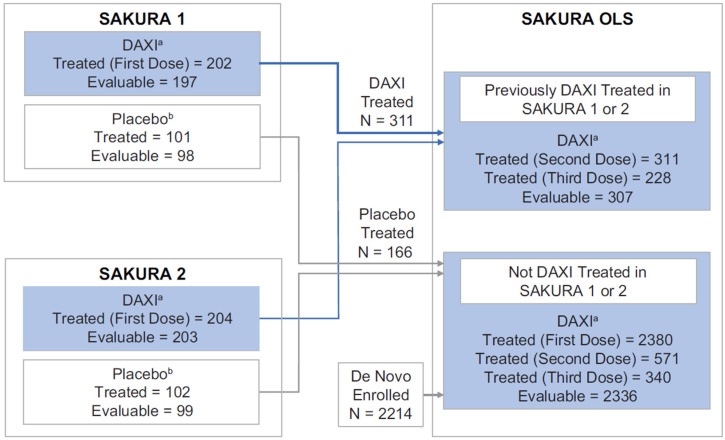
Subject disposition from the three SAKURA trials. ^a^ A total of 2786 subjects received at least 1 DAXI treatment (202 subjects received their first DAXI treatment in SAKURA 1, 204 in SAKURA 2, and 2380 in SAKURA 3); of these, 2737 were evaluable subjects with baseline assessment and at least 1 post-baseline antibody assessment. ^b^ A total of 2823 subjects were exposed to RTP004 (2786 received RTP004 in the DAXI formulation in the three trials, and 203 received RTP004 in placebo in SAKURA 1 or SAKURA 2); of these, 2772 were evaluable subjects with baseline assessment and at least 1 post-baseline antibody assessment. DAXI, DaxibotulinumtoxinA for Injection.

In the SAKURA phase 3 program, 2786 subjects received DAXI 40U, of whom 882 received two treatments and 568 received three treatments. Overall, 2823 subjects were exposed to RTP004, which included 2786 exposed through the active DAXI formulation and an additional 37 subjects who received placebo in SAKURA 1 and 2, did not roll over into the open-label SAKURA 3 study, and thus were exposed only to RTP004 and not DAXI. A total of 914 subjects had at least two exposures and 702 had three exposures to RTP004. The disposition of subjects in the SAKURA studies, including the number of evaluable subjects with baseline and at least one post-baseline antibody assessment (2737 for daxibotulinumtoxinA and 2772 for RTP004; ~98%), is shown in Figure 1. All de novo subjects enrolled in SAKURA 3 received DAXI. Previous BoNTA treatment, which is a risk factor for antibody formation, was noted in 40.1% of subjects in the phase 3 program. Baseline characteristics in subjects exposed to daxibotulinumtoxinA and those exposed to RTP004 are shown in Table 1.

### 2.2. Binding Antibodies to DaxibotulinumtoxinA

At baseline, 12 of 2737 (0.4%) evaluable subjects were found to have binding antibodies to BoNTA. A total of 21 of 2737 subjects (0.8%) developed treatment-induced (*n* = 20) or treatment-boosted (*n* = 1) anti-daxibotulinumtoxinA binding antibodies. The categorization of the antibody response is shown in Table 2. 

**Figure 2:**
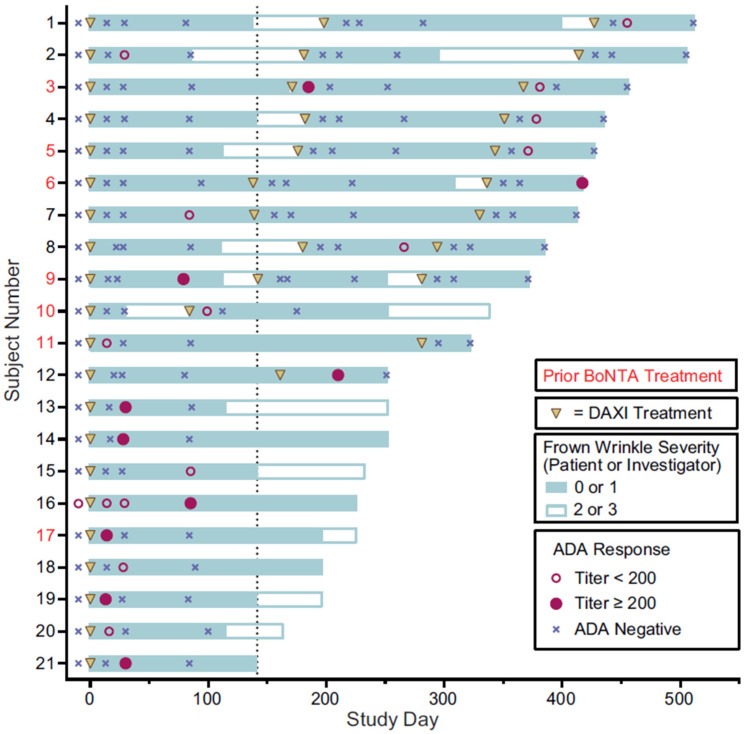
Binding antibodies to daxibotulinumtoxinA and FWS status over time in subjects with treatment-related binding antibodies to daxibotulinumtoxinA. The dotted line represents the median duration of glabellar line response (time to loss of FWS none or mild by both investigator and subject assessment) to DAXI in treatment cycles 1 and 2 in this subgroup. ADA, anti-drug antibody; BoNTA, botulinum neurotoxin type A; DAXI, DaxibotulinumtoxinA for Injection; FWS, Frown Wrinkle Severity (assessed by either subject or investigator).

Most binding antibodies to daxibotulinumtoxinA were transient. In 18 of 19 subjects with further antibody tests after a positive result, their subsequent result(s) were negative. Only two subjects (#3, #16) had more than one anti-daxibotulinumtoxinA binding antibody positive result (Figure 2). Subject #3, who had previous exposure to BoNTAs, had a positive anti-daxibotulinumtoxinA binding antibody result at Week 2 after the second treatment (titer > 1:200), followed by negative results at Week 8 and Week 12. A positive anti-daxibotulinumtoxinA binding antibody test also occurred at Week 2 after the third dose (titer of <1:200), followed by negative results at Week 8 and Week 12. A single subject (#16), who received one DAXI treatment, had treatment-boosted daxibotulinumtoxinA binding antibodies. This subject, who reported no prior exposure to BoNTA, was positive for anti-daxibotulinumtoxinA binding antibodies at baseline with a titer of 1:50, and at two additional time points, with a maximum titer of 1:200 at Week 12 after their DAXI treatment.

Of the 21 subjects with treatment-related anti-daxibotulinumtoxinA antibodies, seven (33.3%) had previously received BoNTA. Overall, 18 of the 21 subjects (all except Subjects #6, #15, and #16; Figure 2) did not test positive for anti-daxibotulinumtoxinA binding antibodies at their final test visit.

### 2.3. Neutralizing Antibodies to DaxibotulinumtoxinA

Serum samples from subjects with a confirmed positive result for anti-daxibotulinumtoxinA binding antibodies were assessed for the presence of neutralizing antibodies using the validated mouse protection assay. No subject with treatment-related anti-daxibotulinumtoxinA binding antibodies tested positive for neutralizing antibodies. 

One subject had neutralizing antibodies at baseline and a consistent titer at additional timepoints after a single treatment, which was categorized as a treatment-unaffected antibody response. This subject, whose last exposure to prior botulinum toxin was approximately 32 months prior to study enrollment, had no clinical response to DAXI and did not report any adverse events (AEs).

### 2.4. Binding Antibodies to RTP004

**Figure 3:**
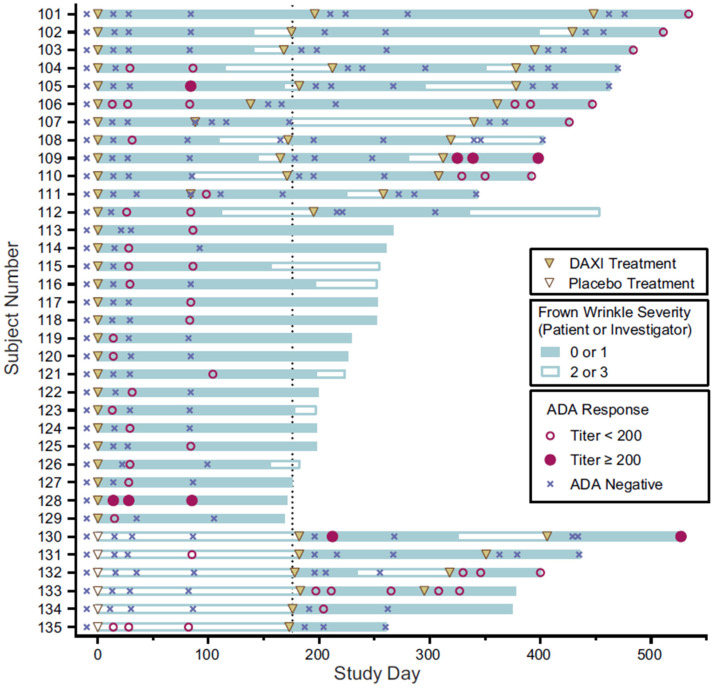
Binding antibodies to RTP004 and FWS status over time in subjects with treatment-induced binding antibodies to RTP004. The dotted line represents the median duration of glabellar line response (time to loss of FWS none or mild by both investigator and subject assessment) to DAXI in treatment cycles 1 and 2 in this subgroup. ADA, anti-drug antibody; DAXI, DaxibotulinumtoxinA for Injection; FWS, Frown Wrinkle Severity (assessed by either subject or investigator).

At baseline, 66 of 2772 (2.4%) evaluable subjects had detectable antibody titers to RTP004. Treatment-related anti-RTP004 binding antibodies were detected in 35 (1.3%) subjects; 17 subjects had one exposure to RTP004, 3 had 2 exposures, and 15 had three exposures (Figure 3). Twenty-three of the 35 subjects had a single positive antibody result with a measured titer of <1:200 which was either shown to be transient by a subsequent negative test result (14 subjects) or was the subject’s final test (nine subjects). No subject had treatment-boosted anti-RTP004 antibodies. 

Four subjects had three consecutive positive antibody titers after their third RTP004 exposure: subject #109 (all titers ≥ 1:200), and subjects #106, #110, and #132 (all titers < 1:200). Subject #133 developed persistent binding antibodies to RTP004 after their second exposure (all titers < 1:200), and subject #130 showed intermittent positivity with titers ≥ 1:200 which were present at the final assessment. Each of these subjects responded to DAXI treatment with no immune-related AEs. No subject developed binding antibodies to both daxibotulinumtoxinA and RTP004.

### 2.5. Clinical Response and Immune-Related AEs in Subjects with Treatment-Induced Binding Antibodies to DaxibotulinumtoxinA or RTP004

Despite the observation that no subjects developed neutralizing antibodies to daxibotulinumtoxinA, the impact of the presence of binding antibodies to daxibotulinumtoxinA or RTP004 on clinical efficacy was nonetheless evaluated. All subjects (100%) with treatment-induced binding antibodies to daxibotulinumtoxinA or RTP004 showed clinical efficacy, achieving a rating of none or mild glabellar line severity on both the Investigator Global Assessment-Facial Wrinkle Severity (IGA-FWS) and Patient Facial Wrinkle Severity (PFWS) scales at Week 4 following each cycle of DAXI treatment. The temporal distribution of binding antibody results, DAXI treatments, and efficacy based on IGA-FWS of none (0) or mild (1) severity, and duration of clinical response for each subject with treatment-related binding antibodies to daxibotulinumtoxinA are shown in Figure 2. A similar representation for subjects with treatment-induced RTP004 binding antibodies is shown in Figure 3.

Duration of clinical benefit was determined for the treatment cycles in which anti-daxibotulinumtoxinA binding antibodies were detected and compared with the duration in treatment cycles in which no anti-daxibotulinumtoxinA binding antibodies were detected. In the 21 subjects with at least one positive test result, the mean duration of efficacy in those study cycles (*n* = 16) with positive binding antibodies to daxibotulinumtoxinA was 142 days (95% CI: 115.0, 282.0), compared with 141 days (95% CI: 111.0, 169.0) for 11 study cycles without positive binding antibodies to daxibotulinumtoxinA. Similarly, for 27 study cycles in which binding antibodies to RTP004 were detected post-treatment, the duration of efficacy (median time to return to moderate or severe glabellar lines following DAXI treatment) was 177.0 days (95% CI: 155.1, 198.1) compared with 138 days (95% CI: 109.7, 159.1) for 26 study cycles with no binding antibodies to RTP004.

No subjects observed to have binding antibodies to either daxibotulinumtoxinA or RTP004 reported the occurrence of immune-related AEs.

## 3. Discussion

This analysis of a large phase 3 clinical development program provides a comprehensive assessment of the immunogenicity of the novel BoNTA formulation, DAXI, and is the first large-scale evaluation of clinical immunogenicity for a PTD-containing formulation. The findings reported herein indicate a low incidence of treatment-induced binding antibodies to daxibotulinumtoxinA (0.7%) and the PTD-containing excipient RTP004 (1.3%). Importantly, no formation of neutralizing antibodies was detected in this study. In subjects with treatment-induced binding antibodies, titers were generally low (<1:200) and no subject had binding antibodies to both daxibotulinumtoxinA and RTP004, suggesting that the RTP004 peptide did not have adjuvant activity. 

Most binding antibodies to daxibotulinumtoxinA were shown to be transient and most positive results to RTP004 were singular with a titer of <1:200. The few subjects who showed persistent anti-RTP004 antibodies demonstrated a clinical response to treatment, suggesting that the binding did not alter the effect of the excipient. Multiple treatments or prior exposure to BoNTA did not show any apparent relationship with the occurrence of binding antibodies to either daxibotulinumtoxinA or RTP004. Additionally, treatment response and duration of response were unaffected by the presence of binding antibodies to daxibotulinumtoxinA or RTP004. There was no evidence that immune-related AEs were associated with the presence of binding antibodies to daxibotulinumtoxinA or RTP004. The one subject who had neutralizing antibodies at baseline and after treatment showed no clinical response to DAXI. Interestingly, the subject had received treatment with another BoNTA up to 32 months before the study, suggesting that the subject had developed an antibody response to the previously administered BoNTA. The presence of baseline reactivity to RTP004 in a small percentage of subjects is believed to reflect interaction of the highly positively charged RTP004 with other serum immunoglobulins rather than being due to the binding of specific antibodies.

The immunogenicity of commercial preparations of BoNTA will be influenced by the dose and re-treatment frequency required for specific indications [19]. In clinical practice, BoNTA is administered in picogram doses for aesthetic indications and in very low nanogram doses for neurologic and urologic indications. The low incidence of binding antibodies shown in the current analysis of DAXI 40U for the treatment of facial wrinkles is expected for BoNTA use in aesthetic indications, and may not be generalizable to dosages used for therapeutic indications [20]. Immunogenicity data from clinical trials of DAXI administered at doses above 40U in patients with cervical dystonia are planned for publication. The efficacy profile of DAXI, with an extended duration of clinical effect, compared with currently approved BoNTAs, could potentially reduce immunogenic risk by allowing less frequent retreatment, thereby reducing the cumulative neurotoxin dose over long-term treatment.

Immunogenicity rates are not directly comparable between BoNTA products owing to differences in multiple factors such as assay sensitivity, sampling procedures, duration of the treatment period, and prior exposure to BoNTA [21]. Nevertheless, the immunogenicity of daxibotulinumtoxinA appears to be at the low end of the range of the incidence of binding antibodies reported for other commercially available BoNTA products across all indications (0–3.6%) [22] and consistent with the low incidence of neutralizing antibodies estimated with aesthetic BoNTA treatment (0.2%) [23]. Overall, the findings demonstrate that the novel DAXI formulation with excipient peptide RTP004 does not impact the immunogenicity rate. 

A limitation of this analysis is the relatively small number of subjects who received multiple doses compared with the longer-term repeated treatment generally seen in clinical practice. However, the large sample size pooled from three SAKURA trials was considered sufficient to assess the absence of daxibotulinumtoxinA neutralizing antibodies. Additionally, we believe this is the first time the three-tier anti-drug antibody testing methodology recommended by the US Food and Drug Administration has been applied to the assessment of BoNTA.

## 4. Conclusions

No neutralizing antibodies to daxibotulinumtoxinA were seen in this rigorous analysis from the large phase 3 program of DAXI, the novel BoNTA formulated with a PTD-containing excipient RTP004, in the treatment of glabellar lines. With repeat local injections of DAXI, the potential for developing binding antibodies to daxibotulinumtoxinA or RTP004 was low and did not lead to treatment failure or immune-related AEs. This first analysis of the risk of antibody formation to a PTD-containing drug product has demonstrated no impact of the peptide excipient, RTP004, on the immunogenicity profile of daxibotulinumtoxinA. These data suggest that the risk of treatment failure or local immune reactions following injection of DAXI is low. However, further evaluation in higher dose indications and with real-world clinical use will provide additional information.

## 5. Materials and Methods

### 5.1. Data Source

This immunogenicity analysis was based on samples obtained from subjects enrolled in the first comprehensive phase 3 clinical program for DAXI, which was for the treatment of glabellar lines. This program consisted of two identically designed double-blind, placebo-controlled, single-dose studies (SAKURA 1 [ClinicalTrials.gov (accessed on 28 November 2022) identifier NCT03014622] and SAKURA 2 [NCT03014635]) and a large open-label safety study (SAKURA 3 [NCT03004248]) evaluating single and up to three repeat treatments [16,24,25]. In these studies, subjects with moderate or severe glabellar lines, as assessed by the investigator and subject on a validated four-point rating scale, received DAXI 40U or placebo (both containing RTP004) at five intramuscular injection sites in the corrugator and procerus muscles of the eyebrow region. For subjects who received more than one treatment, the minimum duration between DAXI treatments was 12 weeks if they returned to baseline severity, with the exception of subjects rolling over from SAKURA 1 and SAKURA 2, who were required to remain in those studies for 24 weeks before rolling over into SAKURA 3. Whole blood specimens for daxibotulinumtoxinA and RTP004 immunogenicity testing were collected at the screening visit for SAKURA 1, 2, and 3, and at Weeks 2, 4, and 12 after each treatment and processed to yield serum samples. The protocols for the three studies were approved by the relevant institutional review board (Advarra, formerly Quorum Review IRB: Quorum File # 31968, 20 October 2016; US Quorum File # 31971, 20 October 2016; Canada Quorum File # 31971CDN, 19 December 2016; US Quorum File # 32089, 23 November 2016; Canada Quorum File # 32089CDN, 19 December 2016) and all subjects provided written informed consent.

### 5.2. Immunogenicity Tests

The presence of reactive serum anti-drug antibodies to daxibotulinumtoxinA and RTP004 was assessed using validated ELISAs. Both ELISA methods detected all human IgG subclasses, IgA, IgE, IgM and to a lesser extent IgD. Briefly, serum samples including controls were diluted 1:50 in assay buffer (2% chicken serum, 0.05% Tween-20 in phosphate buffered saline [PBS]) and incubated on blocked antigen-coated microplate wells (either daxibotulinumtoxinA [Revance Therapeutics, Nashville, TN, USA] or RTP004 [Bachem Americas Inc., Torrance, CA, USA]) on a plate shaker at ambient temperature for approximately 1 h. After the plates were washed, (0.05% Tween-20 in PBS) bound antibodies were detected by incubation for 1 h with Protein-A/G labeled with horseradish peroxidase (Thermo Scientific, Waltham, MA, USA). The plates were washed again and briefly incubated with peroxidase substrate tetramethyl-benzidine (KPL, Milford, MA, USA). After termination of the enzymatic reaction with an acidic stop solution (1N H_2_SO_4_), absorbance was measured by dual-wavelength using 450 nm minus 650 nm (Molecular Devices SpectraMax 340PC with SoftMax Pro v. 6.3 or later, San Jose, CA, USA). As recommended in regulatory guidance documents [1,26], screening, confirmatory, and titer cut points were determined statistically for assays to detect reactive serum antibodies against daxibotulinumtoxinA and RTP004. 

**Figure 4:**
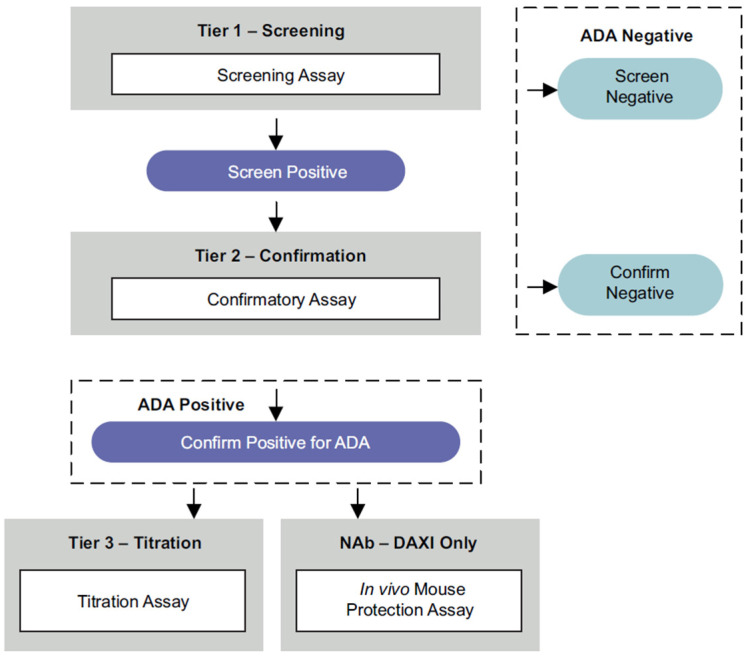
Anti-drug antibody three-tiered testing paradigm comprised (1) screening assay to identify samples positive for reactive antibodies, (2) confirmatory assay for positive samples to confirm binding specificity, and (3) titration assay to estimate antibody titer. Samples confirmed positive for anti-daxibotulinumtoxinA binding antibodies were assessed for the presence of neutralizing antibodies in the validated mouse protection assay. ADA, anti-drug antibody; DAXI, DaxibotulinumtoxinA for Injection; Nab, neutralizing antibody.

A three-tiered testing paradigm consistent with guidance by the US Food and Drug Administration was implemented for in-study testing [26] (Figure 4). It included three distinct testing phases: (1) a screening assay to initially identify test samples as negative or potentially positive for reactive antibodies, (2) a confirmatory assay in which positive samples from step 1 were incubated in the absence and presence of daxibotulinumtoxinA (Revance Therapeutics, Nashville, TN, USA) or RTP004 (Bachem Americas Inc., Torrance, CA, USA) to confirm binding specificity by evaluating the extent of competitive inhibition, and (3) a titration assay in which confirmed-positive serum samples were serially diluted to provide an estimate of the antibody titer. 

**Figure 5:**
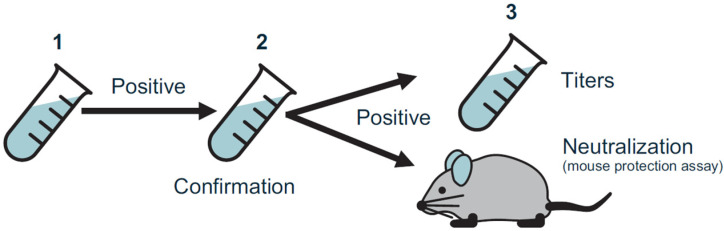
Three-tiered testing paradigm comprised (1) screening assay to identify samples positive for reactive antibodies, (2) confirmatory assay for positive samples to confirm binding specificity, and (3) titration assay to estimate antibody titer. Samples confirmed positive for anti-daxibotulinumtoxinA binding antibodies were assessed for the presence of neutralizing antibodies in the validated mouse protection assay. MPA, mouse protection assay.

Samples confirmed to be positive for the presence of anti-daxibotulinumtoxinA binding antibodies were assessed for the presence of neutralizing antibodies by the validated mouse protection assay [27], whereby serum samples were incubated with daxibotulinumtoxinA (Revance Therapeutics, Nashville, TN, USA) prior to intraperitoneal injection into CD-1 mice (Charles River Laboratories) (Figure 5). Samples were considered to have daxibotulinumtoxinA neutralizing antibodies, if injected mice survived for at least 72 h after the injection. During assay validation, the total purified IgG fraction, derived from a pool of partially purified hyperimmunized rabbit sera containing anti-BoNTA antibodies, was used as a positive control; a concentration of 150 ng IgG fraction per mL of human serum was sufficient to detect daxibotulinumtoxinA-neutralizing activity with this assay. No further testing was conducted on samples confirmed positive for RTP004 as the synthetic peptide has no known intrinsic biological activity. 

### 5.3. Treatment-Relationship of DaxibotulinumtoxinA and RTP004 Immunogenicity

Treatment-related antibody responses included both treatment-induced (negative at baseline and at least 1 antibody positive result post-treatment) or treatment-boosted (positive at baseline with an increase in antibody titer post-treatment to at least 4 times the baseline value). Subjects with a positive antibody result at baseline and no increase in titer during the treatment follow-up were classified as treatment-unaffected. The baseline binding antibody assessments prior to the first DAXI or placebo treatment in SAKURA 1 and SAKURA 2 were the baseline values for determining treatment-induced or treatment-boosted antibody status, including those for subjects who rolled over to SAKURA 3. The baseline for newly enrolled subjects in SAKURA 3 was the screening visit. 

### 5.4. Effect of Immunogenicity on Efficacy and Safety

The effect of the presence of anti-drug binding antibodies on the efficacy of DAXI in improving glabellar line severity was assessed using the validated, four-point photonumeric IGA-FWS and PFWS scales. Both scales rate glabellar line severity from none (no wrinkles; score of 0) to severe (deep wrinkles; score of 3). Clinical benefit was measured as the achievement of none or mild glabellar line severity as assessed by the investigator using the IGA-FWS at each treatment cycle. The duration of clinical benefit was defined as the time to return to moderate or severe glabellar line severity on both the IGA-FWS and PFWS and was assessed in treatment cycles 1 and 2, in which subjects were followed for up to 36 weeks. The duration was not assessed in cycle 3 because subjects were followed for only 12 weeks after the third treatment to characterize safety outcomes. The duration of clinical benefit was summarized with point estimates of median duration and two-sided, 95% confidence intervals using log-log transformation by the summary group. Statistical analyses were performed using SAS version 9.4 (SAS Institute, Cary, NC, USA) or R version 3.6.0 (R Foundation for Statistical Computing, Vienna, Austria).

The impact of anti-drug antibodies on the safety of DAXI was assessed by examining the occurrence of immune-related AEs reported following each treatment.

## Figures and Tables

**Table 1:** Baseline characteristics of subjects exposed to daxibotulinumtoxinA and to RTP004 in the SAKURA phase 3 program.

	Exposed to DaxibotulinumtoxinA (*n* = 2786)	Exposed to RTP004 (*n* = 2823)
**Female, %**	88.5	88.5
**Age, years, mean (SD)**	49.4 (11.3)	49.4 (11.3)
**Race, %**		
White	89.2	89.2
Black/African American	5.0	4.9
Asian	2.7	2.7
Other	3.1	3.2
**Time since last BoNTA, months, median (range)**	18.1 (5.3–319.9)	18.0 (5.3–319.9)
**IGA-FWS at maximum frown, %**		
Moderate	62.1	62.1
Severe	37.9	37.9
**PFWS at maximum frown, *n* (%)**		
Moderate	56.7	56.7

BONTA, botulinum toxin type A; IGA-FWS, Investigator Global Assessment-Frown Wrinkle Severity; PFWS, Patient Frown Wrinkle Severity; SD, standard deviation.

**Table 2:** Immune responses to daxibotulinumtoxinA and RTP004 in evaluable subjects by treatment in SAKURA 1, 2, and 3.

Treatment Cycle # (Evaluable Subjects, DaxibotulinumtoxinA /RTP004) ^a^	Response Type	Exposed to DaxibotulinumtoxinA (*n* = 2737)	Exposed to RTP004 (*n* = 2772)
Cycle 1 (*n* = 2737/2770)	Negative	2713 (99.1%)	2680 (96.8%)
	Treatment-induced	12 (0.4%)	24 (0.9%)
	Treatment-unaffected	11 (0.4%)	66 (2.4%)
	Treatment-boosted	1 (0.04%)	0
Cycle 2 (*n* = 873/906)	Negative	864 (99.0%)	865 (95.5%)
	Treatment-induced	4 (0.5%)	4 (0.4%)
	Treatment-unaffected	5 (0.6%)	37 (4.1%)
	Treatment-boosted	0	0
Cycle 3 (*n* = 566/696)	Negative	557 (98.4%)	659 (94.7%)
	Treatment-induced	5 (0.9%)	10 (1.4%)
	Treatment-unaffected	4 (0.7%)	27 (3.9%)
	Treatment-boosted	0	0
Overall ^b^ (*n* = 2737/2772)	Negative	2705 (98.8%)	2671 (96.4%)
	Treatment-induced	20 (0.7%)	35 (1.3%)
	Treatment-unaffected	11 (0.4%)	66 (2.4%)
	Treatment-boosted	1 (<0.1%)	0

^a^ Placebo contained RTP004; therefore, RTP004 exposure for placebo rollover subjects began in SAKURA 1 and SAKURA 2, while their daxibotulinumtoxinA exposure started after they rolled over to SAKURA 3. Hence, the number of evaluable subjects was different between daxibotulinumtoxinA and RTP004 in each treatment cycle, ^b^ Subjects with a positive response in more than one cycle are counted once in the overall category.

## Data Availability

Deidentified data are available on request from Revance Therapeutics, Inc.

## References

[1] Vandivort T.C., Horton D.B., Johnson S.B. (2020). Regulatory and strategic considerations for addressing immunogenicity and related responses in biopharmaceutical development programs. J. Clin. Transl. Sci..

[2] Dolimbek B.Z., Aoki K.R., Steward L.E., Jankovic J., Atassi M.Z. (2007). Mapping of the regions on the heavy chain of botulinum neurotoxin A (BoNT/A) recognized by antibodies of cervical dystonia patients with immunoresistance to BoNT/A. Mol. Immunol..

[3] Atassi M.Z., Dolimbek B.Z., Jankovic J., Steward L.E., Aoki K.R. (2011). Regions of botulinum neurotoxin A light chain recognized by human anti-toxin antibodies from cervical dystonia patients immunoresistant to toxin treatment. The antigenic structure of the active toxin recognized by human antibodies. Immunobiology.

[4] Krebs K.M., Lebeda F.J. (2008). Comparison of the structural features of botulinum neurotoxin and NTNH, a non-toxic accessory protein of the progenitor complex. Botulinum J..

[5] Göschel H., Wohlfarth K., Frevert J., Dengler R., Bigalke H. (1997). Botulinum A toxin therapy: Neutralizing and nonneutralizing antibodies—Therapeutic consequences. Exp. Neurol..

[6] Glogau R.G., Waugh J.M. Preclinical Transcutaneous Flux Experiments using a Macromolecule Transport System (MTS) Peptide for Delivery of Botulinum Toxin Type A. Proceedings of the Annual Meeting of the American Academy of Dermatology.

[7] Smyth T., Oliyai C., Joshi A. Stabilizing Effect of RTP004 on Non-Specific Surface Adsorption in Drug Product Manufacturing of Daxibotulinumtoxina (DAXI). Proceedings of the TOXINS 2019.

[8] Weisemann J., Rummel A., Oliyai C., Too P., Joshi A. Novel Peptide Excipient RTP004 Enhances the Binding of Botulinum Neurotoxin A Cell Binding Domain Hc to Rat Brain Synaptosomes. Proceedings of the TOXINS 2019.

[9] Yin L., Masuyer G., Zhang S., Zhang J., Miyashita S.I., Burgin D., Lovelock L., Coker S.F., Fu T.M., Stenmark P. (2020). Characterization of a membrane binding loop leads to engineering botulinum neurotoxin B with improved therapeutic efficacy. PLoS Biol..

[10] Jankovic J., Comella C., Hauser R.A., Patel A.T., Gross T.M., Rubio R.G., Vitarella D. ASPEN-1. A Phase 3 Trial Evaluating the Efficacy, Duration of Effect, and Safety of DaxibotulinumtoxinA for Injection in the Treatment of Cervical Dystonia. Proceedings of the TOXINS 2021.

[11] Allergan (2020). BOTOX (onabotulinumtoxinA) for Injection, for Intramuscular, Intradetrusor, or Intradermal Use. Prescribing Information. https://media.allergan.com/actavis/actavis/media/allergan-pdf-documents/product-prescribing/20190620-BOTOX-100-and-200-Units-v3-0USPI1145-v2-0MG1145.pdf.

[12] Comella C.L., Jankovic J., Shannon K.M., Tsui J., Swenson M., Leurgans S., Fan W., Dystonia Study Group (2005). Comparison of botulinum toxin serotypes A and B for the treatment of cervical dystonia. Neurology.

[13] Field M., Splevins A., Picaut P., Van der Schans M., Langenberg J., Noort D., Foster K. (2018). AbobotulinumtoxinA (Dysport^®^), OnabotulinumtoxinA (Botox^®^), and IncobotulinumtoxinA (Xeomin^®^) neurotoxin content and potential implications for duration of response in patients. Toxins.

[14] Carruthers J., Solish N., Humphrey S., Rosen N., Muhn C., Bertucci V., Swift A., Metelitsa A., Rubio R.G., Waugh J. (2017). Injectable daxibotulinumtoxinA for the treatment of glabellar lines: A phase 2, randomized, dose-ranging, double-blind, multicenter comparison with onabotulinumtoxinA and placebo. Dermatol. Surg..

[15] Bertucci V., Solish N., Kaufman-Janette J., Yoelin S., Shamban A., Schlessinger J., Snyder D., Gallagher C., Liu Y., Shears G. (2020). DaxibotulinumtoxinA for Injection has a prolonged duration of response in the treatment of glabellar lines: Pooled data from two multicenter, randomized, double-blind, placebo-controlled, phase 3 studies (SAKURA 1 and SAKURA 2). J. Am. Acad. Dermatol..

[16] Fabi S.G., Cohen J.L., Green L.J., Dhawan S., Kontis T.C., Baumann L., Gross T.M., Gallagher C.J., Brown J., Rubio R.G. (2021). DaxibotulinumtoxinA for Injection for the treatment of glabellar lines: Efficacy results from SAKURA 3, a large, open-label, phase 3 safety study. Dermatol. Surg..

[17] Allergan (2019). BOTOX Cosmetic (onabotulinumtoxinA) for Injection, for Intramuscular Use. Prescribing Information. https://media.allergan.com/actavis/actavis/media/allergan-pdf-documents/product-prescribing/20190626-BOTOX-Cosmetic-Insert-72715US10-Med-Guide-v2-0MG1145.pdf.

[18] Merz (2019). XEOMIN (incobotulinumtoxinA) for Injection, for Intramuscular or Intraglandular Use. Prescribing Information. https://www.xeominaesthetic.com/wp-content/uploads/2019/05/XEOMIN-Full-Prescribing-Information-including-MedGuide.pdf.

[19] Albrecht P., Jansen A., Lee J.I., Moll M., Ringelstein M., Rosenthal D., Bigalke H., Aktas O., Hartung H.P., Hefter H. (2019). High prevalence of neutralizing antibodies after long-term botulinum neurotoxin therapy. Neurology.

[20] Walter U., Mühlenhoff C., Benecke R., Dressler D., Mix E., Alt J., Wittstock M., Dudesek A., Storch A., Kamm C. (2020). Frequency and risk factors of antibody-induced secondary failure of botulinum neurotoxin therapy. Neurology.

[21] Naumann M., Boo L.M., Ackerman A.H., Gallagher C.J. (2013). Immunogenicity of botulinum toxins. J. Neural. Transm..

[22] Bellows S., Jankovic J. (2019). Immunogenicity associated with botulinum toxin treatment. Toxins.

[23] Rahman E., Alhitmi H.K., Mosahebi A. (2021). Immunogenicity to botulinum toxin type A: A systematic review with meta-analysis across therapeutic indications. Aesthet. Surg. J..

[24] Carruthers J.D., Fagien S., Joseph J.H., Humphrey S.D., Biesman B.S., Gallagher C.J., Liu Y., Rubio R.G., Sakura T. (2020). DaxibotulinumtoxinA for Injection for the treatment of glabellar lines: Results from each of two multicenter, randomized, double-blind, placebo-controlled, phase 3 studies (SAKURA 1 and SAKURA 2). Plast. Reconstr. Surg..

[25] Green J.B., Mariwalla K., Coleman K., Ablon G., Weinkle S.H., Gallagher C.J., Vitarella D., Rubio R.G. (2021). A large, open-label, phase 3 safety study of DaxibotulinumtoxinA for Injection in glabellar lines: A focus on safety from the SAKURA 3 study. Dermatol. Surg..

[26] US Food and Drug Administration (2019). Immunogenicity Testing of Therapeutic Protein Products—Developing and Validating Assays for Anti-Drug Antibody Detection. Guidance for Industry. https://www.fda.gov/regulatory-information/search-fda-guidance-documents/immunogenicity-testing-therapeutic-protein-products-developing-and-validating-assays-anti-drug.

[27] Hatheway C., Dang C., Jankovic J., Halett M. (1994). Immunogenicity of the Neurotoxins of Clostridium Botulinum. Therapy with Botulinum Toxin. Neurological Disease and Therapy.

